# Personal air pollution exposure and metals in the nasal epithelial lining fluid of COPD patients

**DOI:** 10.1088/2752-5309/acbbe5

**Published:** 2023-03-01

**Authors:** Hilary L Zetlen, Anna Stanley Lee, Lina Nurhussien, Wendy Sun, Choong-Min Kang, Antonella Zanobetti, Mary B Rice

**Affiliations:** 1 Division of Pulmonary, Critical Care & Sleep Medicine, Beth Israel Deaconess Medical Center, Boston, MA, United States of America; 2 Department of Environmental Health, Harvard T.H. Chan School of Public Health, Boston, MA, United States of America

**Keywords:** COPD, nasal epithelial lining fluid, air pollution exposure, metals exposure, exposure assessment, PM_2.5_, black carbon

## Abstract

Sampling of the nasal epithelial lining fluid is a potential method to assess exposure to air pollution within the respiratory tract among high risk populations. We investigated associations of short- and long-term particulate matter exposure (PM) and pollution-related metals in the nasal fluid of people with chronic obstructive pulmonary disease (COPD). This study included 20 participants with moderate-to-severe COPD from a larger study who measured long-term personal exposure to PM_2.5_ using portable air monitors and short-term PM_2.5_ and black carbon (BC) using in-home samplers for the seven days preceding nasal fluid collection. Nasal fluid was sampled from both nares by nasosorption, and inductively coupled plasma mass spectrometry was used to determine the concentration of metals with major airborne sources. Correlations of selected elements (Fe, Ba, Ni, Pb, V, Zn, Cu) were determined within the nasal fluid. Associations between personal long-term PM_2.5_ and seven day home PM_2.5_ and BC exposure and nasal fluid metal concentrations were determined by linear regression. Within nasal fluid samples, concentrations of vanadium and nickel (*r* = 0.8) and lead and zinc (*r* = 0.7) were correlated. Seven day and long-term PM_2.5_ exposure were both associated with higher levels of copper, lead, and vanadium in the nasal fluid. BC exposure was associated with higher levels of nickel in the nasal fluid. Levels of certain metals in the nasal fluid may serve as biomarkers of air pollution exposure in the upper respiratory tract.

## 1. What this study adds

This pilot study investigates the detection of metals within the nasal fluid as a potential technique to assess personal air pollution exposure among adults with chronic obstructive pulmonary disease (COPD). This study is the first of its kind examining short- and long-term exposure to air pollution exposure and levels of combustion-related metals in the nasal fluid of non-occupationally exposed adults. We found that COPD patients with higher personal exposure to particulate matter (PM) had higher levels of vanadium in the nasal fluid, which is linked to fuel oil combustion. Higher exposure to black carbon (BC), a component of PM_2.5_ more specific to combustion, was also associated with nickel in the nasal fluid. Our investigation demonstrates that analysis of nasal fluid may be a promising, minimally invasive technique for assessing and characterizing personal exposure to air pollution in susceptible populations.

## 2. Introduction

Exposure to air pollution is associated with increased risk of exacerbation, emergency department visits, and hospitalization in individuals with COPD [1–3]. There is a need to develop clinical techniques to better assess air pollution exposures among COPD patients in order to identify those at higher risk who may benefit from interventions to reduce exposure. Analysis of nasal epithelial lining fluid (NELF) samples is a promising minimally invasive method to identify elements from ambient pollution sources that may have deposited in the upper respiratory tract.

Sampling of the nasal cavity is an established method for assessing biomarkers of inflammation in response to inhaled exposures [4–6]. Specific components of air pollution**—**e.g. metallic constituents—are also detectable in nasal samples. Occupational studies of heavily-exposed workers have found that metal concentrations in nasal lavage fluid (iron [Fe], nickel [Ni], and others) are associated with fine PM_2.5_ exposure [7]. However, this association has not been examined in non-occupationally exposed adults.

To evaluate if metal concentrations in the nasal lining fluid are associated with higher exposure to PM and combustion-related pollution, we conducted a pilot study nested within the long-term Study of Pollution and COPD Exacerbation (SPACE) [8]. This study employs a novel method of sampling NELF using an absorbent fibrous matrix inserted into the nares. This technique has been used effectively for analysis of biomarkers of inflammation in response to inhaled exposures [9]. We hypothesized that metal concentrations in the NELF of community-dwelling adults with COPD may serve as tracers of air pollution exposure.

## 3. Methods

A total of 20 participants with GOLD Stage II–IV moderate-to-severe COPD who were enrolled in the SPACE in the Boston area agreed to participate in a NELF pilot study; further details of recruitment have been described elsewhere [8].

For this pilot study, we measured indoor air pollution exposure in the home as well as long-term exposure using a portable air monitor (PAM). Short-term indoor PM_2.5_ was measured with cascade impactor samplers [10] in the home for the seven days preceding nasal sampling. Gravimetric filter samples were collected using Teflon filters and PM_2.5_ concentration for the seven day sampling period was determined by measurement of pre- and post-sampling filter weights and sampler flow rate as previously described [10]. BC was determined from the PM_2.5_ fraction by the optical absorbance measurement using the SootScan OT21 Transmissometer (Magee Scientific, Berkeley, CA, USA), which is a cost-effective, non-destructive method that quantifies filter particles using optical measurements at the wavelength of 880 nm [11]. Participants also measured personal long-term PM_2.5_ exposure by PAM for 4 months over a 12 month period. We estimated long-term PM_2.5_ exposure by taking the average daily measurement over all four months of data collection for each participant.

At the end of the seven day indoor air sampling period, NELF was sampled once in both nares using an absorbent fibrous matrix (Leukosorb medium) soaked in sterile saline solution [9]. 16 out of 20 nasal samples were collected during fall and winter months. Following a previously described procedure [9], Ergonomic Leukosorb strips were inserted in both nostrils for 2 min, then removed and placed in sterile vials for storage.

For the NELF metals analysis, elements were selected based on previously reported associations with indoor and outdoor pollution sources, including motor vehicle exhaust and road dust (barium [Ba], copper [Cu], Fe, lead [Pb], zinc [Zn]) [12–14], oil combustion (Ni, vanadium [V]) [15, 16] and cooking (Fe, Zn) [12, 13, 17, 18].

The concentration of these metals was determined in the NELF using inductively coupled plasma mass spectrometry [19] at the Wisconsin State Laboratories. Each of the elements used in the analysis had minimal detection on blank fibrous matrices. Samples from both nares were averaged for each participant and reported as the average concentration (ng per nasosorption strip). To evaluate whether metals from the same sources were present within NELF, we assessed correlations between metal concentrations in the NELF samples using Spearman correlations.

Using linear regression models, we examined if seven day PM_2.5_ and BC in the home and long-term (four month average) PM_2.5_ by PAM were associated with per-person averaged nasal fluid metal concentrations. We also examined models using log-transformed nasal fluid metal concentrations. The primary models were unadjusted. We also evaluated models adjusted for participant sex and age at baseline visit. Additionally, we performed a sensitivity analysis to determine whether nasal metal concentration may serve as a predictor of air pollutant exposure using linear regression models with nasal metal concentration as the primary predictor and log-transformed pollutant concentrations as the outcome.

## 4. Results

**Figure 1.**
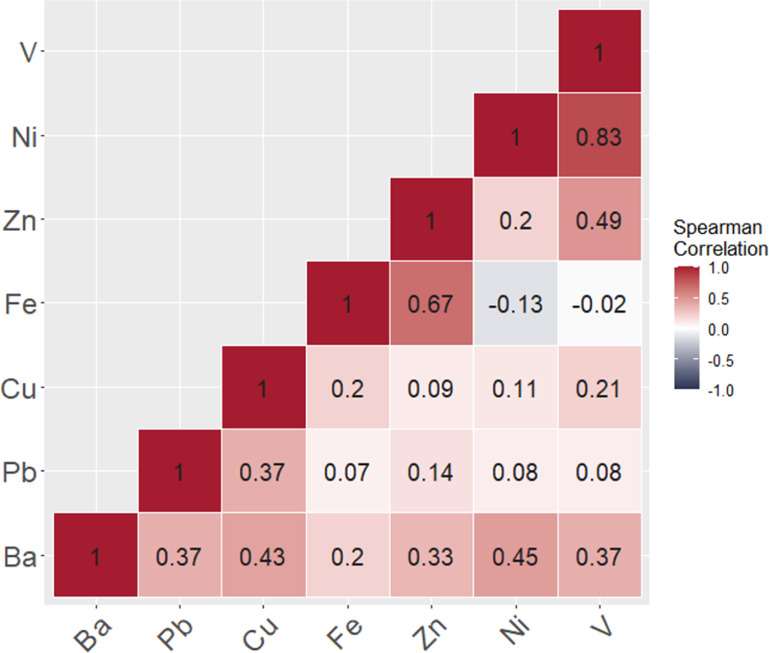
Correlations between metal concentrations within the nasal epithelial lining fluid.

The median (interquartile range [IQR]) seven day PM_2.5_ and BC, and long-term PM_2.5_ concentrations were 6.1 *µ*g m^−3^ (11.3), 0.3 *µ*g m^−3^ (0.3), and 9.5 *µ*g m^−3^ (4.5), respectively. Short- and long-term PM_2.5_ concentrations were highly correlated (Pearson *r* = 0.811, p *<*0.001). Within the nasal fluid, several metals with airborne sources had moderate to strong correlations: V and Ni (*r* = 0.8) and Fe and Zn (*r* = 0.7) (figure 1).

In unadjusted linear regression models using non-transformed nasal metals concentrations, both seven day and long-term PM_2.5_ were associated with higher levels of Cu, Pb and V in the nasal fluid. For example, an IQR difference in seven day PM_2.5_ was associated with 29.8 ng higher Cu (95% CI, 15.3–44.4), 0.2 ng higher Pb (95% CI, 0.1–0.4), and 0.2 ng higher V (95% CI, 0.0–0.4) levels in the nasal fluid (table 1). Seven day BC exposure was associated with higher levels of Ni (1.8 ng, 95% CI 0.4–3.3) and V (0.3 ng, 95% CI −0.1 to 0.6) in the nasal fluid. Results were unchanged with adjustment for age and gender, and linear regression models using log-transformed nasal fluid metal concentrations demonstrated similar, though less precise, positive associations across the aforementioned exposure categories (table S2). In the sensitivity analysis examining associations between nasal metal concentration and log-transformed pollutant concentration as the outcome, positive associations persisted between nasal Cu, V and Pb concentrations and both short- and long-term PM_2.5_, and between nasal Ni and short-term BC (table S3).

**Table 1.** Associations between personal exposure to particulate air pollution and levels of metals in the nasal fluid (ng per nasosorption strip)^a^.

Element	Difference per IQR increase in seven day PM_2.5_ ng (95% CI)	Difference per IQR increase in seven day black carbon ng (95% CI)	Difference per IQR increase in long-term PM_2.5_ ng (95% CI)
Fe	−10.9 (−45.6, 23.9)	−6.5 (−49.5,36.6)	−6.6 (−33.1, 19.8)
Ba	−0.1 (−0.8, 0.6)	−0.2 (−1.1, 0.6)	−0.03 (−0.6, 0.5)
Ni	0.6 (−0.8, 1.9)	**1.8 (0.4, 3.3)**	0.2 (−0.8, 1.2)
Pb	**0.3 (0.1, 0.5)**	−0.01 (−0.3, 0.3)	**0.2 (0.1, 0.4)**
V	**0.3 (0.03, 0.6)**	0.3 (−0.1,0.6)	**0.2 (0.04, 0.4)**
Zn	−47.0 (−219.9, 126.0)	−115.8 (−322.2, 90.7)	−16.7 (−148.9, 115.5)
Cu	**29.8 (15.3, 44.4)**	0.9 (−24.5, 26.3)	**20.4 (8.6, 32.1)**

^a^ All differences per interquartile range (IQR) increment in exposure: seven day PM_2.5_ = 11.28 *μ*g m^−3^, seven day BC = 0.30 *μ*g m^−3^, long-term PM_2.5_ = 4.52 *μ*g m^−3^. CI = confidence interval. **p < 0.05.**

## 5. Discussion

In this study of adults with moderate-to-severe COPD with relatively low exposure to particulate air pollution in the home, we found that some metals in the nasal fluid, in particular vanadium, copper, and lead, were associated with higher personal exposure to airborne PM. BC exposure, which is more specific to motor vehicle emissions, was associated with higher levels of nickel in NELF, but was not associated with levels of other elements in NELF (i.e. Ba, Pb, Cu, Fe) that have been attributed to motor vehicle emissions when measured in the air [12–14]. Our findings suggest that nasosorption is a promising technique for identifying individuals who are more highly exposed to pollution in their daily lives.

Characterizing metals in nasal fluid can provide insight into the most significant indoor and outdoor airborne emissions sources to which at-risk patients may be exposed. The correlations observed among metallic air pollution constituents in NELF samples correspond to potential emissions sources. Notably, vanadium and nickel are common constituents of outdoor emissions from oil combustion, while zinc and iron are frequently found in indoor emissions from cooking, as well as outdoor sources such as motor vehicle exhaust and road dust. Additionally, while heavy metal concentrations in blood and urine have previously been associated with acute and chronic PM exposure levels [20], samples derived directly from airway epithelium have not been examined in non-occupationally exposed groups. Our method utilizes samples taken from the nasal epithelium, which interfaces directly with the environment. Nasosorption may be a more relevant assessment of exposures that can directly affect pulmonary disease, including COPD.

Specific metals identified in our analysis may have a mechanistic role in the pathophysiology of human respiratory disease. Vanadium is easily transported in fine PM and is found in emissions from oil combustion [16]. In animal studies, inhaled vanadium exposure has been shown to have immunomodulatory effects in cell-cell interactions in the thymus, spleen, and lymph nodes [21–23], and has also been associated with lung injury in animal models [24]. Copper is found in emissions from multiple sources, including road dust [13, 17]. Several studies suggest an association between serum copper levels and adverse respiratory outcomes, such as decreased pulmonary function and increased risk of COPD, especially in males [25, 26]. Proposed mechanisms include increased activation of lung fibroblasts as well as increased neutrophilic inflammation in lungs exposed to copper [27, 28]. Finally, lead is found in emissions from vehicles and road dust, among other sources [15]. Both ambient and occupational lead exposures are associated with lower pulmonary function in adults [29, 30].

Our study evaluated a new nasal biomarker assessment method for environmental health research and has several limitations. As this is a small pilot study, the focus was not on the statistical significance or the magnitude of the associations observed, but rather the patterns of associations between exposure to particulate pollutants (PM_2.5_, BC) and metals in the nasal fluid. Given the limited scope of this pilot project, we did not evaluate elemental concentrations in air filter data—obtaining these data in future studies would provide valuable insight into the correlations between nasal and air concentrations of metals of interest. Future analyses including metals concentration data from air filters would also allow for additional insights into potential sources for specific metals in participants’ environments. For example, while vanadium is a useful marker of residual fuel oil combustion when measured in the air, further research is needed to determine whether vanadium is a biomarker specific to this exposure when measured in the nasal fluid. Overall, our sample size was small, with a focus on exploring methodology for future air pollution biomarker research. Undertaking a larger study integrating more exposure data—i.e. outdoor air pollution exposure, elemental analysis of air pollution—would allow for better assessment of elemental groupings (e.g. through factor analysis) and linkages to potential environmental sources. Additionally, we are unable to assess to what extent the metallic elements detected in the nasal fluid are derived from non-inhaled exposures (e.g. diet). The PM fraction (2.5 *µ*m) captured in PAMs and ambient air monitors may be distinct from the fraction filtered by the nares, where a broader range of particle sizes can deposit [31]. Finally, while we collected personal PM_2.5_ exposure longitudinally, our study includes a single assessment of NELF metal concentrations and is therefore cross sectional. We cannot confirm if week-to-week variability in the seven day averages of PM_2.5_ or BC is associated with within-person variability in nasal fluid metal concentrations.

While there is a need for further research, nasosorption is a promising minimally invasive technique for assessment of metallic constituents of inhaled particle exposure in high risk groups such as our study population with COPD. We found that levels of some pollution-related metals in nasal fluid, in particular vanadium, copper, and lead, may indicate higher short- and long-term personal exposure to airborne PM in COPD patients. Analysis of nasal fluid as a means to identify high risk patients with higher exposure to air pollution merits further investigation.

## Data Availability

The data that support the findings of this study are available upon reasonable request from the authors.
